# Delayed Strangulated Right Diaphragmatic Hernia after Retroperitoneal Tumor Resection with Hepatectomy: A Case Report

**DOI:** 10.70352/scrj.cr.25-0704

**Published:** 2026-04-08

**Authors:** Naoki Kawahara, Wataru Takayama, Koji Morishita

**Affiliations:** Trauma and Acute Critical Care Center, Institute of Science Tokyo Hospital, Tokyo, Japan

**Keywords:** acquired diaphragmatic hernia, acute care surgery, iatrogenic disease, strangulated diaphragmatic hernia

## Abstract

**INTRODUCTION:**

Delayed iatrogenic diaphragmatic hernia after abdominal surgery is an uncommon complication; however, cases occurring after hepatectomy are relatively more commonly reported in the literature. Such hernias often develop after a significant latency period, but may progress to life-threatening emergencies, including bowel strangulation. Here, we report a rare case of delayed right diaphragmatic hernia following hepatectomy.

**CASE PRESENTATION:**

A man in his 70s, with a history of right hepatectomy for retroperitoneal liposarcoma 4 years prior, presented with acute abdominal pain. CT revealed a strangulated right diaphragmatic hernia with intestinal ischemia. The hernia orifice, which had enlarged by approximately 10 mm/year compared with prior imaging over the preceding 2 years, measured 55 mm on CT but was found to be an 80-mm defect intraoperatively. Emergency surgery confirmed extensive bowel necrosis, requiring large intestinal resection and primary repair. The patient developed short bowel syndrome and was discharged on POD 24.

**CONCLUSIONS:**

Post-hepatectomy diaphragmatic hernias may enlarge and be underestimated on imaging. Early surgery and close follow-up are recommended in such cases, as delayed intervention can result in life-threatening complications.

## INTRODUCTION

Acquired diaphragmatic hernia is an uncommon condition, with iatrogenic injury constituting a particularly rare etiology.^1,2)^ Although liver surgery, most often right hepatectomy, is a potential cause of iatrogenic diaphragmatic hernia,^2–4)^ its occurrence is rare, with reported incidence rates ranging from 0.16% to 2.3%.^2–6)^ The clinical presentation may remain vague and delayed for months to years.^2,7)^ This extended latent period is thought to occur because a small, unrecognized iatrogenic defect can gradually enlarge owing to the persistent pressure gradient between the abdominal and thoracic cavities.^6)^ Progressive enlargement of the hernia orifice may increase the risk of incarceration involving a greater volume or extent of the abdominal organs, thereby predisposing the patient to more severe complications. Herein, we report a case of right diaphragmatic hernia that developed following retroperitoneal tumor resection with right hepatectomy.

## CASE PRESENTATION

A man in his 70s was referred to our hospital with upper abdominal pain and vomiting that had developed the previous day. Four years earlier, the patient underwent laparotomy for resection of a retroperitoneal liposarcoma, which included right hepatectomy, right adrenalectomy, and right nephrectomy. According to the operative records obtained from the previous institution, a minor iatrogenic injury to the right diaphragm occurred during tumor dissection using an energy device. The defect was recognized intraoperatively and repaired with continuous nonabsorbable 2-0 sutures. Details regarding the exact size of the defect and depth of the injury were not documented in the operative report. The patient’s postoperative course was uneventful, with no thoracic or abdominal symptoms reported during follow-up. However, CT performed 1 year and 11 months before presentation revealed a right diaphragmatic hernia measuring 35 mm in diameter. Subsequent CT scans demonstrated that the hernial orifice had gradually enlarged to 53 mm 1 month before presentation and was managed conservatively.

Upon admission, his vital signs were as follows: Glasgow Coma Scale, E3 V5 M6; temperature, 36.0°C; pulse rate, 106/min; blood pressure, 122/87 mmHg; respiratory rate, 23 breaths/min; and SpO_2_, 96% on room air. A reverse L-shaped surgical scar from the previous surgery, extending from the upper abdomen to the right flank, was observed. Physical examination revealed upper abdominal pain without marked tenderness or peritoneal irritation.

**Fig. 1:**
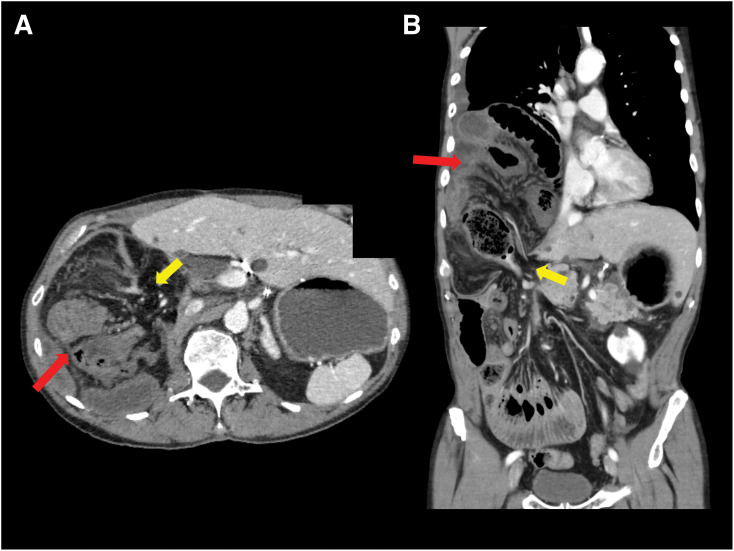
CT scan from the referring hospital. The yellow arrows denote the defect in the right diaphragm, and red arrowheads indicate the herniated ascending colon and small intestine within the right thoracic cavity. (**A**) Axial slice. (**B**) Coronal slice.

Laboratory investigations showed a white blood cell count of 15000/μL, C-reactive protein level of 0.26 mg/dL, impaired renal function (blood urea nitrogen 27.2 mg/dL, creatinine 1.30 mg/dL), elevated amylase level of 1091 U/L, and elevated lactate level of 3.9 mmol/L. CT revealed a 55-mm hernia orifice in the right diaphragm, through which the ascending colon and the majority of the small intestine had herniated (**Fig. 1**).

The herniated bowel showed poor contrast enhancement, suggesting intestinal necrosis.

**Fig. 2:**
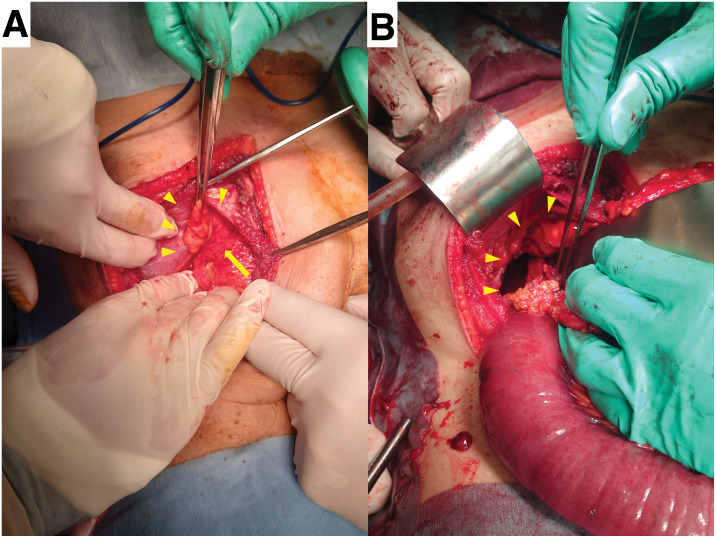
Intraoperative photographs. (**A**) Intraoperative photograph showing the abdomen opened via a right subcostal incision. The yellow arrowheads indicate the site at which the abdominal contents herniated into the right thoracic cavity through a diaphragmatic defect, and the yellow arrow indicates the incarcerated abdominal contents. (**B**) Intraoperative photograph taken after reduction of the herniated contents. The yellow arrowheads indicate the hernia orifice in the right diaphragm.

**Fig. 3:**
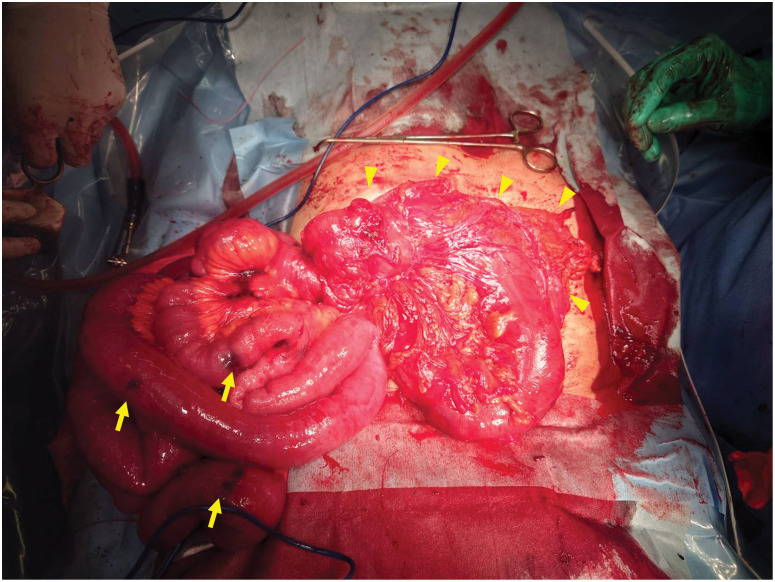
The intestine and omentum immediately after releasing the strangulation. The intestine shows extensive edema, thinning, and focal necrosis. The yellow arrows indicate the necrotic areas of the intestine, and the yellow arrowheads indicate the omentum.

The patient underwent emergency surgery immediately. Considering the patient’s previous reverse L-shaped incision scar and the need to secure a clear view of the right diaphragm, emergency laparotomy was performed through a right subcostal incision. We identified a hernia orifice in the right diaphragm, through which segments of the intestine and greater omentum had herniated (**Fig. 2A**). After reducing the incarcerated intestine and greater omentum, we confirmed that the diaphragmatic defect measured approximately 80 mm, which was larger than that measured on preoperative imaging (**Fig. 2B**). More than half of the small intestine, extending up to the ascending colon, had herniated through the defect. The unusually extensive herniation was considered to be due to lax retroperitoneal fixation of the ascending colon, which likely increased colonic mobility and facilitated subsequent incarceration through the diaphragmatic defect. The bowel segment extracted through the hernial orifice was partially necrotic and diffusely thinned, with marked congestion and edema, and scattered black discoloration suggesting segmental necrosis (**Fig. 3**). Although peristalsis was observed in a limited portion of the bowel after releasing the strangulation, overall peristaltic activity was markedly reduced, and minimal improvement was noted during the observation period. Considering the presence of multiple separate segments suspicious of necrosis, we judged that an extensive resection was required to remove the affected bowel in a single segment to prevent delayed ischemic complications. We performed extensive resection of the ischemic bowel, from 100 cm distal to the ligament of Treitz to the transverse colon, and completed an end-to-end anastomosis. The resected bowel measured 170 cm, and the residual small bowel was approximately 100 cm in length from the ligament of Treitz. The defect was closed with interrupted 1-0 absorbable suture material, as a tension-free approximation was achieved intraoperatively. A drain was placed in the right lower abdomen beneath the right diaphragm, the abdomen was closed, and the procedure was completed.

His postoperative course was complicated by diarrhea and dehydration, leading to a diagnosis of short bowel syndrome requiring antidiarrheal therapy. After bowel function stabilization, the patient was discharged on POD 24. At the time of writing this report, no recurrence of the diaphragmatic hernia has been observed.

## DISCUSSION

This case demonstrates the critical outcome of a right diaphragmatic hernia and massive bowel resection due to strangulation. Based on the previous operative record indicating that the iatrogenic right diaphragm injury obtained during the procedure was repaired intraoperatively, the hernia was considered a delayed iatrogenic complication that progressively enlarged over the years after the initial procedure. Clinical manifestations of diaphragmatic hernias range from nonspecific respiratory or gastrointestinal symptoms to life-threatening hypoxemia and acute, irreducible hernia when strangulation or cardiopulmonary compression occurs.^1,8)^ However, in a previous review of post-hepatectomy diaphragmatic hernias, only a small proportion of patients (6.9%) were incidentally identified on postoperative follow-up imaging, whereas the vast majority were diagnosed after developing clinical manifestations, such as abdominal pain, anorexia, or bowel obstruction.^2)^ In our case, a right diaphragmatic hernia was identified 2 years post-surgery and showed progressive enlargement, although the patient remained asymptomatic until 4 years postoperatively. Furthermore, the hernia orifice measured 55 mm on preoperative CT but was found to be an 80-mm defect intraoperatively. This suggests that the orifice progressively enlarges over time and that its true size can be underestimated on imaging, since accurate evaluation of the right diaphragmatic hernia is often hindered by respiratory motion artifacts and similar contrast between the liver and diaphragm.^8,9)^ Although definitive guidelines for the management of asymptomatic acquired diaphragmatic hernias are lacking, surgical repair has been suggested to prevent serious complications.^1,2,10)^ Our case suggests that defect size may be underestimated on imaging and can enlarge over time, supporting consideration of early intervention in select patients.

Surgical approach selection is a key consideration in acquired diaphragmatic hernia, with multiple options including thoracic or abdominal approaches, with or without mesh reinforcement.^1,11)^ In emergency settings, particularly in hemodynamically unstable patients or when bowel obstruction or ischemia is suspected, open surgery is often required.^1,10)^ In the present case, laparotomy was selected because of ileus and suspected bowel necrosis; however, in less urgent situations, the operative strategy should be individualized based on the patient’s condition, degree of contamination, defect size, and tissue tension. For repair of a diaphragmatic defect, both primary closure and mesh reinforcement have advantages and limitations. Primary suture repair avoids the use of foreign material and is preferable in contaminated fields, as it reduces the risk of mesh-related infection or the need for explantation.^1,8)^ A limitation of primary closure is that excessive tension during repair may predispose to recurrence, particularly in larger defects.^1)^ In contrast, mesh reinforcement allows tension-free repair and may reduce recurrence in large defects; however, it carries risks of infection, erosion, and adhesions, particularly in emergency settings or when bowel resection is performed.^1,8,10)^ The use of prosthetic mesh is generally recommended in the surgical repair of diaphragmatic hernia when tension-free primary closure is not feasible, such as in cases with a hernia diameter >8 cm or defects exceeding 20–30 cm².^1)^ In the present patient, although the hernia orifice measured 80 mm and the defect was relatively large, the diaphragm was sufficiently extensible to permit tension-free primary closure; therefore, simple sutured repair was performed. In light of the emergency setting and associated bowel resection, mesh reinforcement was avoided due to the risk of postoperative infection in a potentially contaminated surgical field. Currently, there is no consensus regarding the optimal suture technique for primary diaphragmatic defect repair.^11–13)^ Classically, diaphragmatic defects have been repaired using interrupted nonabsorbable mattress sutures.^1)^ In the present patient, interrupted sutures were selected because a tension-free approximation was achievable based on intraoperative findings. In cases with larger defects or anticipated closure under tension, tension-reducing strategies extrapolated from abdominal wall reconstruction and plastic surgery, such as purse-string suturing, continuous small-bite techniques, or staged closure, may be considered.^14,15)^ However, robust evidence supporting their use in this setting is lacking, and no consensus has been established regarding the optimal approach. Therefore, further investigation is warranted.

In our patient, progressive enlargement of the hernia orifice and loose retroperitoneal fixation of the ascending colon led to extensive bowel herniation and necrosis. To our knowledge, no previous report has documented an incarcerated iatrogenic diaphragmatic hernia requiring bowel resection of such magnitude, resulting in short bowel syndrome. This case highlights the importance of considering early surgical intervention once a right diaphragmatic hernia is identified, as the actual defect may be larger than it appears on imaging, even in asymptomatic patients. To facilitate timely management, a high index of suspicion is required for at-risk patients presenting with acute gastrointestinal symptoms. Future large-scale studies are needed to clarify the optimal timing of surgery for acquired diaphragmatic hernias. Imaging-based risk stratification may help predict disease progression and support the development of standardized management strategies.

## CONCLUSIONS

This case reaffirms that a delayed iatrogenic right diaphragmatic hernia, although rare, is a potentially devastating complication of hepatectomy. A long and variable asymptomatic period necessitates a high index of suspicion. Early recognition, careful follow-up, and timely surgical repair are essential to prevent catastrophic outcomes, including massive intestinal resection and development of short bowel syndrome.
